# Health seeking behavior among individuals presenting with chronic cough at referral hospitals in Uganda; Missed opportunity for early tuberculosis diagnosis

**DOI:** 10.1371/journal.pone.0217900

**Published:** 2019-06-06

**Authors:** Winters Muttamba, Willy Ssengooba, Bruce Kirenga, Rogers Sekibira, Simon Walusimbi, Achilles Katamba, Moses Joloba

**Affiliations:** 1 Makerere University Lung Institute, College of Health Sciences, Makerere University, Kampala, Uganda; 2 Department of Medical Microbiology, College of Health Sciences, Makerere University, Kampala, Uganda; 3 School of Medicine, College of Health Sciences, Makerere University, Kampala, Uganda; University of Otago, NEW ZEALAND

## Abstract

**Background:**

Tuberculosis (TB) is the 9^th^ leading cause of death from a single infectious agent. Patients live in a complex health care system with both formal and informal providers, and it is important that a TB diagnosis is not missed at the first interaction with the health care system. In this study, we highlight the health seeking behavior of patients and missed opportunities for early TB diagnosis for which interventions could be instituted to ensure early TB diagnosis and prompt TB treatment initiation.

**Methods:**

This study was nested in a cross-sectional study that assessed the accuracy of different Xpert MTB/Rif implementation strategies in programmatic settings at the referral hospitals in Uganda. We documented the symptom profile of presumptive TB patients and assessed the health seeking behavior of those with chronic cough by calculating proportion of patients that visited each type of health facility and further calculated the odds of being TB positive given the type of health facility initially visited for consultation.

**Results:**

A total of 1,863 presumptive TB patients were enrolled of which 979 (54.5%) were male, and 1795 (99.9%) had chronic cough. A total of 1352 (75.4%) had previously sought care for chronic cough, with 805 (59.6%) seeking care from a public health facility followed by private health facility (289; 21.4%). Up to 182 (13.5%) patients visited a drug store for chronic cough. Patients whose first contact was a private health facility were more likely to have a positive GeneXpert test (adjOR 1.4, 95% CI: 1.0–1.9; p = 0.047).

**Conclusions:**

Chronic cough is a main symptom for many of the presumptive TB patients presenting at referral hospitals, with several patients having to visit the health system more than once before a TB diagnosis is made. This suggests the need for patients to be thoroughly evaluated at first interface with the health care system to ensure prompt diagnosis and treatment initiation. Improved TB diagnosis possibly with the GeneXpert test, at first contact with the health care system has potential to increase TB case finding and break the transmission cycle in the community.

## Background

Tuberculosis (TB) is the 9^th^ leading cause of death from a single infectious agent, ranking above Human Immune deficiency virus (HIV) [1]. The 2017 WHO global TB report lists Uganda as a high HIV/TB burden country. In 2016, Uganda notified 44816 TB cases [2], and data from the TB prevalence survey put the prevalence and incidence rates at 253/100,000 and 234/100,000 respectively [3]. Uganda has a decentralized laboratory health system with laboratory services decentralized up to lower level health facilities. The country has up to 1,500 diagnostic and treatment units (DTUs) and as early as 2010, Xpert MTB/Rif technology was rolled out in the country, with up to 115 machines installed in 105 health facilities [4]. Loop holes in the health care system are some of the reasons for delayed and missed TB diagnosis. Studies have shown that patients first identified as having TB in community surveys had previously attended health services on a number of occasions with symptoms but had never been diagnosed [5].

Prompt diagnosis and early initiation of treatment remain key strategies for TB prevention and control. Diagnostic delays have been documented both at patient and health system levels [6, 7]. Factors that could lead to delay in TB diagnosis include initial visitation to a low level public healthcare facility, private practitioner consultation, or visit to a traditional healer, poverty, female gender, alcoholism and substance abuse, history of immigration, low educational status, low awareness of TB, self-prescription and stigma [5, 8–10]. Studies done in Uganda, Ghana and India found health systems as the biggest contributor of delayed TB diagnosis [8, 9, 11, 12]. Health facility delay was strongly associated with failure to perform sputum microscopy and was more common among private healthcare practitioners and rural public health facilities [11].

Patients live in a complex health care system with both formal and informal providers and it is important that a TB diagnosis is not missed at first contact with the health care system. Missed opportunities at first contact with the healthcare system do not only jeopardize the wellbeing of the patients but also have the potential to increase and sustain TB transmission in the community. In this study, we document the heath seeking behaviors of patients with chronic cough (cough of more than 2 weeks) and highlight the missed opportunities for early TB diagnosis for which interventions could be instituted to ensure early TB diagnosis and prompt treatment initiation.

## Methods

### Design and study setting

This study was nested in a cross-sectional study that assessed the accuracy of different Xpert MTB/Rif implementation strategies in programmatic settings at referral hospitals in Uganda [13]. We consecutively enrolled presumptive TB patients (18 years and above) at five referral hospitals in Uganda. Four of the hospitals (Mbale, Arua, Mbarara and Lacor) are regional referral hospitals serve as referral units for lower level health units in the region each with an average bed capacity of 450 beds. The other referral hospital (Mulago) is a national referral hospital that provides services to Kampala's population and surrounding districts plus upcountry referrals and has up to 1500 beds.

A presumptive TB patient was defined as a patient with chronic cough (at least 2 weeks), significant weight loss (more than 3Kgs), persistent fevers (more than 3 weeks) and excessive night sweats (more than 3 weeks). The participants were both HIV positive and negative adults visiting these study sites for consultation, with some having previously sought care from other health facilities. We excluded patients that were too ill to be interviewed. The study sites were well spread across the country with some sites serving cross border populations by virtue of them being near or in border towns. The study was conducted between October 2015 and August 2016.

All presumptive TB patients able to provide sputum had their sputum samples tested on the Xpert MTB/RIF assay. TB positive patients were linked into care and started on TB treatment as per the national guidelines. Xpert MTB/RIF assays were done at the study sites according to standard procedures and as have been detailed in previous work[13].

### Data collection

Data were collected through use of an interviewer administered questionnaire that had been previously piloted. Interviewers were trained on how to fill the questionnaire. The questionnaire covered sociodemographic as well as health seeking history of the respondents. The interviewers ensured all questions were asked before the interview could end.

### Statistical analysis

Data were double entered into an electronic database (Epidata Version 2.0 /2007) and where there were discrepancies, these were resolved by cross checking with the source documents (raw data). Data were exported to Stata v13 (Stata Corp, College Station TX, USA) for analysis. We used the exact binomial method for calculating 95% confidence intervals.

We documented the symptom profile of presumptive TB patients and assessed the health seeking behavior of those with chronic cough by calculating the proportion of patients that visited each type of health facility and further calculated the odds of being TB positive on GeneXpert given the type of health facility initially visited for consultation. Also assessed was the proportion of patients asked for a TB test at each of the facilities visited even when the patients presented with chronic cough. Variables at bivariate analysis with P-Value < 0.2 were added to a logistic regression model in a backward elimination method. Variables with P-value < 0.05 were considered statistically significant.

### Ethics statement

Approval was obtained from Makerere School of Public health IRB (HDREC 093) and national approval granted by Uganda National Council for Science and Technology (HS-1904). All participants gave written informed consent.

## Results

### Characteristics of the study population

A total of 1,863 presumptive TB patients were enrolled, of which 66 patients had incomplete data. Only 1797 patients who had complete demographic data were included for analysis. Table 1 summarizes the demographic characteristics of the respondents (presumptive TB patients), and the study site distribution was as follows: Mbale 200 (11.1%), Arua 429 (23.9%), Mbarara 235 (13.1%), Lacor 246 (13.7%) and Mulago 687 (38.2%). The males were 979 (54.5%), 1,072 (57.5%) were in the age group of 18–38 years, subsistence farming was the dominant occupation group; 475 (26.4%) and 1065 (59.2%) respondents were married.

**Table 1:** Socio-demographic characteristics of adults enrolled in health care services with respiratory symptoms (n = 1797).

Variable	Category	Frequency	Percentage
Sex	Male	979	54.5
	Female	818	45.5
Age	18–38	1072	57.5
	39–59	588	31.6
	>60 years	203	10.9
Residence	Urban	989	55.0
	Rural	808	45.0
Marital status	Single	389	21.6
	Married	1065	59.2
	Separated	184	10.2
	Divorced	43	2.4
	Widowed	88	4.9
	Other	28	1.6
Occupation	Unemployed	277	15.4
	Housewife	105	5.8
	Subsistence farmer	475	26.4
	Market vendor	148	8.2
	Builder	78	4.3
	Health worker	10	0.6
	Business	355	19.8
	Civil servant	67	3.7
	Others	282	15.7

### Health seeking behavior of the presumptive TB patients with chronic cough

**Fig 1:**
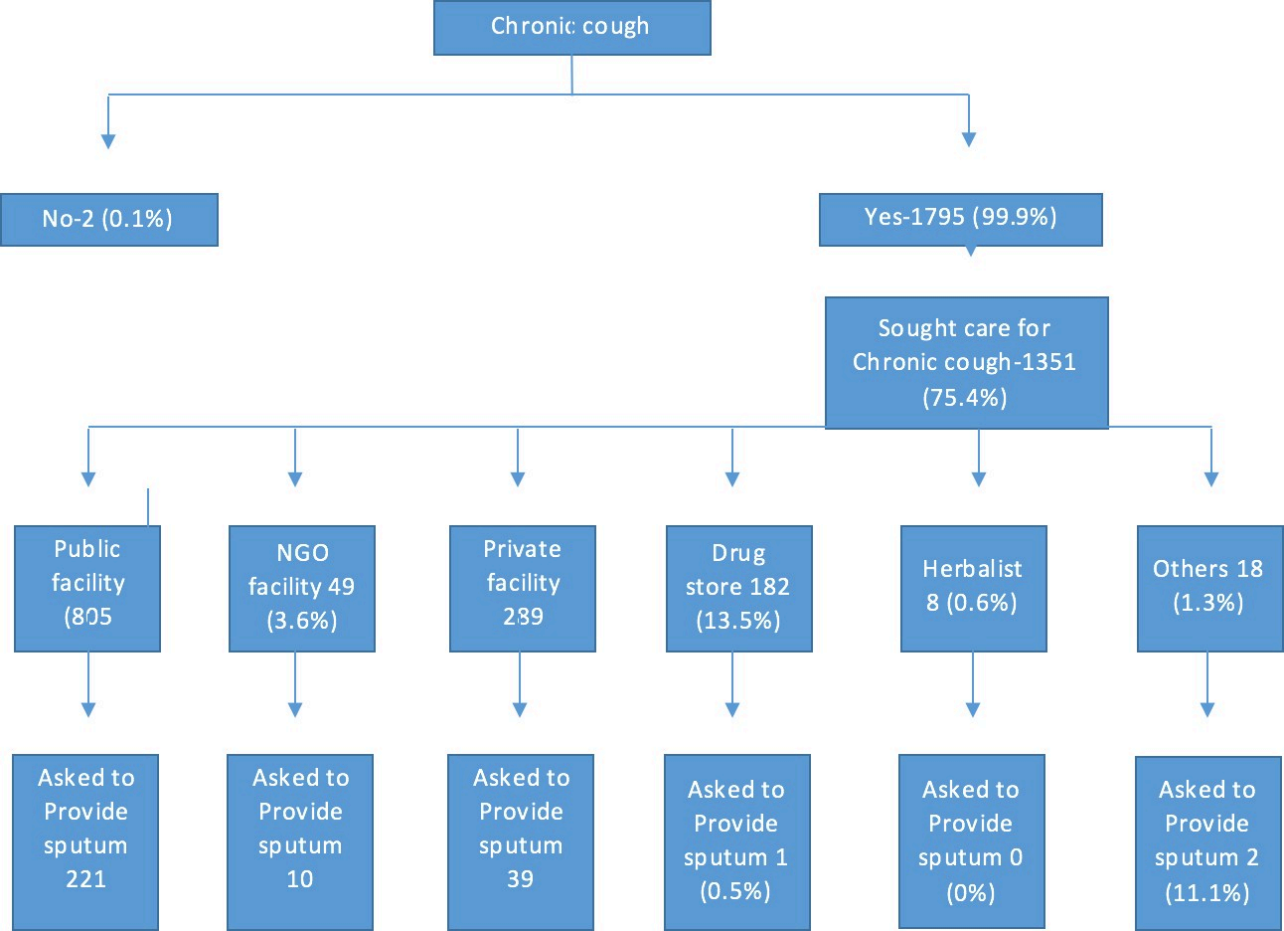
Health Seeking behavior for the presumptive TB patients with chronic cough.

The health seeking behavior of the respondents with chronic cough is represented in Table 2 and Fig 1. Fig 1 also shows the proportion of patients at each of the facilities visited that were asked to avail a sputum sample when they presented with chronic cough. Of the respondents, 1795 (99.9%) had chronic cough as a symptom. Of these, 1351 (75.2%) had previously sought care for the chronic cough with 805 (59.6%) seeking care from a public health facility followed by 289 (21.4%) visiting a private health facility. Up to 182 (13.5%) patients visited a drug store for the chronic cough. Of the patients that consulted from a public health facility, 221 (27.5%) were asked to provide a sputum sample, while 39 (13.5%) of those that consulted from a private health facility were asked to provide a sputum sample.

**Table 2:** Table showing health facilities initially visited by patients with chronic cough (n = 1351).

Health facility visited following chronic cough	Frequency	Percentage
Public health facility	805	59.6
Non-Governmental Organization (NGO) health facility	49	3.6
Private health facility	289	21.4
Drugstore/Pharmacy	182	13.5
Herbalist	8	0.6
Others*	18	1.3

*Others = (friends, Ayurveda, nutritional supplements)

### GeneXpert results of the respondents given health facility initially visited

The GeneXpert results of the respondents based on the health facilities initially visited are represented in Table 3 below. Over a quarter (30%) of the respondents were TB positive by GeneXpert. Of the respondents that consulted from herbalists for the chronic cough, 50% were GeneXpert positive, while 36.1% of those that consulted from a private health facility tested positive on GeneXpert.

**Table 3:** GeneXpert results of the respondents given health facility initially visited (n = 1316).

Health facility	Total	GeneXpert Positive n (%)
Public	781	218 (27.9)
NGO	48	14(29.2)
Private	280	101(36.1)
Drug store	182	52(28.6)
Herbalist	8	4(50)
Others*	17	6(35.3)
Total	1,316	395(30.0)

*Others = (friends, Ayurveda, nutritional supplements)

### Probability of being GeneXpert positive given the health facility initially visited

The odds of being found GeneXpert positive given the health facility initially visited are presented in Table 4. The odds of being GeneXpert positive having initially visited a private health facility were 1.4 times higher (AdjOR 1.4, 95% CI: 1.0–1.9; p = 0.047) than when the facility initially visited was a public health facility.

**Table 4:** Odds of being GeneXpert positive based on health facility initially visited.

Health facility	Un adjOR (95% CI)	P-Value	AdjOR* (95% CI)	P-Value
Public	Reference		Reference	
NGO	1.1 (0.6–2.0)	0.851	1.0 (0.5–2.0)	0.971
Private	**1.5 (1.1–1.9)**	**0.011**	**1.4 (1.0–1.9)**	**0.047**
Drugstore	1.0 (0.7–1.5)	0.859	1.0 (0.7–1.1)	0.844
Herbalist	2.6 (0.6–10.4)	0.182	2.0 (0.5–4.2)	0.353
Others	1.4 (0.5–3.9)	0.505	1.4 (0.5–4.2)	0.520

*Adjusted for age, gender, education level, bio smoke exposure, occupation

## Discussion

This study done among referral hospitals shows that a significant proportion of presumptive TB patients presents with chronic cough as one of the key symptoms. Despite the fact that these patients reside in a high burden setting, they are usually misdiagnosed for TB during their initial presentation at the heath facilities. Moreover, even with this attempt of care seeking given their chronic cough, most of them were not evaluated for TB during the initial care seeking visit. We document a significant number of these patients who end up being diagnosed with TB using the GeneXpert test at the referral health facilities. Our study further shows that a number of these participants had initially presented to at least one health facility with chronic cough before TB diagnosis was made. This presents a missed opportunity for early TB diagnosis in a high TB/HIV burden setting.

This multiple healthcare contact following signs and symptoms could lead to delays in TB diagnosis [14, 15]. Provider shopping has been found to be a patient related factor contributing to TB diagnostic delays [16]. Studies done have shown that despite several visits to clinics with signs and symptoms suggestive of TB disease, patients still go un diagnosed. This could be due to lack of diagnostic awareness among health workers, as well as atypical presentation [17].

Shortage of health workers particularly laboratory technicians could also lead to failure to diagnose patients for TB at their initial visit [17]. The failure to diagnose TB in these facilities could also be due to absence of diagnostics which could be further complicated by stock outs of consumables e.g. sputum mugs, reagents among others. There is a need to further decentralize TB services including roll out of the GeneXpert technology to lower facilities to aid in diagnosis of TB patients. GeneXpert has been found to be more sensitive than microscopy [13].

In this study, chronic cough was the most common symptom among the presumptive TB patients as has been documented elsewhere [16, 18]. However in a survey done in Zambia, cough was documented a secondary symptom to chest pain[19], probably due to the fact that this was a community prevalence survey which tends to find TB in patients without cough.

Our study further found that most of the patients sought initial care at a public health facility. This is probably because of the decentralized public health system setting in Uganda which goes as low as the village level. Furthermore, this could also be due to the fact that most of the respondents were subsistence farmers, and services in public health facilities are free of charge. This is in agreement with studies done in rural South Africa and Zambia which found that TB patients often presented initially to public hospitals [19, 20]. Interestingly, the 21% of the participants in our study who sought initial TB care services at the private health facility had 1.5 times chance of being diagnosed with TB using GeneXpert. This could be due to the fact that patients in private health facilities are usually misdiagnosed for TB as a result of low suspicion index coupled with lack of TB diagnostics at the private health facilities. Studies have shown that patients who seek care from a private health facility incur delays in the diagnostic pathway [21, 22]. There are many reasons patients resort to private health facilities such as drug stores/pharmacies including other informal health providers. These include convenience, affordability and social and cultural effects [23]. Pharmacies and drug stores lack adequately trained professionals, provide advice for common symptoms which is not in accordance with guidelines and inappropriately prescribe medicines. There are also gaps in knowledge and provider practice [23, 24]. Important to note, patients that sought care from herbalists were 2.6 times more likely to have a positive GeneXpert result, although not statistically significant. Herbs could be an easy option for most financially handicapped TB presumptive patients as they are a cheaper option.

The main sample for TB diagnosis is sputum, however despite patients presenting to a health facility with chronic cough, most of them were never asked to avail a sputum sample. This further presents a missed opportunity for early TB diagnosis and could facilitate continued community TB transmission [20]. In a Zambian TB survey, only a small proportion of patients (12.1%) that had visited a facility for consultation were asked for a sputum sample [19]. Provider related factors have been found to contribute to delays in TB diagnosis [16], and to further complicate TB control [25]. This thus calls for a high index of suspicion for TB in health facilities if early TB diagnosis is to be realized.

## Limitations

We were unable to document any other visits between the initial visit to the health care provider and the study site. This would have documented a complete pathway the study patients went through before a diagnosis was made. Furthermore, it would have helped us estimate the time between onset of cough and diagnosis to be able to document the magnitude of diagnostic delay. We were also unable to ascertain the level of the public health facility initially visited and also ascertain the test results for those patients that had been asked to provide a sputum sample on initial consultation. The level of the public health facility would have helped us understand whether the facility had the necessary diagnostics or not, while by understanding the results from the sputum, we would have been able to document whether a diagnostic test was done or not when sputum sample was requested.

## Conclusion

Chronic cough is a main symptom for majority of the presumptive TB patients presenting at referral hospitals, with several patients having to visit the health system more than once before having a diagnosis. This suggests the need for patients to be thoroughly evaluated at first interface with the health care system to ensure prompt diagnosis and treatment initiation. Improved TB diagnosis possibly with the GeneXpert test, at first contact with the health care system has potential to increase TB case finding and break the transmission cycle in the community. Patients who first seek TB care from private facilities are 1.5 times more likely to have a positive sputum GeneXpert test result compared to those who first seek care in public health facilities. This stresses the need to strengthen the Public Private Partnerships in Uganda in order to improve TB control.

## Supporting information

S1 Data1 (DTA)

S1 CRF (PDF)
