# Supplementary material for: Health seeking behavior among individuals presenting with chronic cough at referral hospitals in Uganda; Missed opportunity for early tuberculosis diagnosis
Source: PLoS One. 2019 Jun 6;14(6):e0217900. doi: 10.1371/journal.pone.0217900 (PMC6553765; doi:10.1371/journal.pone.0217900)
Supplement: S1 CRF — (PDF) [file pone.0217900.s002.pdf]

THE EAST AFRICAN PUBLIC HEALTH LABORATORY NETWORKING (EAPHLN) PROJECT

Operations Research Component

EVALUATION OF IMPACT OF NEW TUBERCULOSIS DIAGNOSTICS SCALE-UP ON TUBERCULOSIS DIAGNOSIS AND OUTCOMES

Study Identification Number ☐☐☐☐☐☐ Hospital Registration Number ☐☐☐☐☐

Patient Initials ☐☐☐

SOCIO-DEMOGRAPHIC

1. Study Site *(circle one)*	2. Which department is the client coming from *(circle one)*	3. Marital Status *(circle one)*	
1. Mbale<br>2. Arua<br>3. Mbarara<br>4. Gulu<br>5. Mulago	1. OPD<br>2. Self-referral<br>3. Inpatient<br>4. Special clinic e.g. HIV Clinic, Diabetes clinic<br>5. Other	1. Single<br>2. Married<br>3. Separated<br>4. Divorced<br>5. Widowed<br>6. Other__________	**4. DATE**: ___/______ /_____(dd /mm/yyyy)<br>**5. Age:**____________<br>**6. Sex:** 1. Male ☐ 2. Female ☐

7. Residence *(circle one)* 1. Urban 2. Rural
8. Is this your home or you are visiting because of treatment? *(circle one)* 1. Home 2. Visiting for treatment
9. Village ____ 10. Parish: ___________________________________

11. Sub county___________________ 12. District________________13. Phone Contact: _________________________
14. Approximate distance from home to this health facility in Kms_________________

15. Is this your nearest health facility? 1.Yes 2.No

16. If No, why did you decide to come to this health facility
   1. Referred by a health worker ☐
   2. No TB diagnosis at the nearest health facility ☐
   3. Other__________________________________

17. Were you referred to this health facility? 1.Yes 2. No

18. If yes, which facility referred you?
   1. Government hospital ☐
   2. Private clinic ☐
   3. Drug store/Pharmacy ☐
   4. Health Centre ☐
   5. Other__________________

RISK FACTORS

19. Are you exposed to biomass smoke (charcoal, wood, industrial etc)? 1. Yes 2. No

20. Yes, state hours of exposure per day__________________

21. Occupation *(circle one)*
1. Unemployed
2. Housewife
3. Peasant farmer
4. Market vendor
5. Builder
6. Health Worker
7. Business
8. Civil servant
9. Farmer
10. Other. Specify ______________

22. Highest level of Education *(circle one)*
1. None
2. Incomplete primary
3. Complete primary
4. Incomplete secondary
5. Complete secondary
6. Tertiary

23. Average daily income____________

24. Do you have a family member who has or has ever had TB? 1. Yes 2. No

25. Do you know anyone who has TB? 1. Yes 2. No

26. If Yes, how many___________________

27. Do you share a bedroom with someone who is not your spouse? 1. Yes 2. No

PAST MEDICAL HISTORY

28. In the past 12 months, is there any member of your household who was diagnosed with TB? *(Circle one)*

1. Yes 2. No

29. Do you smoke tobacco? *(Circle one)*

1. Yes 2. No 3. Unknown

30. If Yes, how often

1. Daily 2. Less than daily (not every day)
3. Unknown

31. Duration of smoking (current and past in years)

____

32. Number of tobacco pieces/sticks smoked per day_________________________

33. Do you drink alcohol? 1. Yes 2. No

(if yes answer questions 34-37)

34. If Yes, how often?

1. Daily
2. Less than daily (not every day)
3. Unknown

35. Duration of alcohol consumption (current and past in years) ______________________

36. How many drinks per day___________________

37. How many drinks per week___________________

38. History of Diabetes *(Circle one)* 1. Yes 2. No

39. RBS (mmol/l)___________________

40. History of Cancer 1. Yes 2. No

41. Have you been previously treated for TB? [Probe for long respiratory illness, TB symptoms, long courses of treatments including injections of more than a month and chest x-rays to help patient remember) *(Circle one)*

1. Yes 2. No

42. If Yes

a) When were you treated?_________________ (state the year of treatment)

b) How many times have you been treated? __________________ (times)

c) Outcome of the last treatment according to the patient*(circle one that applies)*

1. Cured
2. Failed
3. Defaulted
4. Treatment completed
5. Unknown

KNOWLEDGE ON TB

43. Did you ever think that the cough you have could be TB? *(Circle one)* 1. Yes 2.No

44. If Yes; how did you know?*(Circle one)*

1. Relative
2. Health worker
3. Friend
4. Media (Radio, TV, Newspaper etc)

45. Before today, did you know there is a laboratory test available for diagnosis of TB?*(Circle one)*

1. Yes 2. No

46. If yes, which of these did you hear about?*(Circle one)*

1. Microscopy
2. Culture
3. GeneXpert

47. Do you know how TB is spread?*(Circle one)*

1.Yes 2.No 3.Don’t know

48. If Yes, how?*(Circle one)*

1. Cough
2. Contact
3. Sharing utensils
4. Other_____________

49. Do you think TB is curable?*(Circle one)*

1.Yes 2.No 3.Don’t know

CLINICAL DATA

50. Weight_________ (kgs)	51. Height _________ (cm) 52. BCG Scar 1. Present 2. Absent

SYMPTOMS

Symptom	Duration in days	
53. Chronic cough	1. Yes [ ]	2. No
54. Fever	1. Yes [ ]	2. No
55. Weight loss	1. Yes [ ]	2. No
56. Excessive Night sweats	1. Yes [ ]	2. No
57. Dyspnea	1. Yes [ ]	2. No
58. Chest Pain	1. Yes [ ]	2. No
59. Blood stained sputum	1. Yes [ ]	2. No
60. Other Specify _______________	[ ]	

HIV STATUS

61. Do you know your HIV status? 1. Yes 2. No *(if no, go to question 5)*
62. If yes, what is your HIV status? 1. HIV positive 2. HIV Negative
63. If no send for HIV test and record results 1. HIV positive 2. HIV Negative
64. If HIV positive are you on antiretroviral drugs? 1. Yes 2. No
65. If yes, duration in months ______________________

BEHAVIOUR REGARDING COUGH

66. **Did you seek medical care for your chronic cough?***(Circle one)* *1.*Yes 2. No

67. If **Yes** **(a) Where did you first seek care***(Circle)*
1. Public health facility
2. NGO facility
3. Private health facility
4. Drug store/Pharmacy
5. Herbalist
6. Other_________________

(b) What service did you receive? *(Circle all that apply)*
1. Given medicine
2. Chest Xray
3. Asked to provide sputum
4. Referred elsewhere for care
5. Physical exam/Consultation
6. Other___________________

68. **If no, what is the main reason for not seeking care?** *(Circle)*
1. Self-treatment
2. Didn't think it was a serious illness
3. Ignore
4. Cost
5. Distance
6. Long waiting time
7. My church/mosque leader stopped me
8. I was using herbs
9. I was undergoing spiritual treatment by a traditional healer
10. I feared that my friends/relative would know I have TB (stigma)
11. Other_________________________

DELAYS

69. **Indicate period in days taken for each of the following;**

70. Time taken before client presented to any health care facility after the onset of symptoms

....

71. Time between presentation to this diagnostic health facility (study site) and sputum specimen submission.

....

72. Time taken from sputum submission to issuing of results

....

73. Time taken before initiation of treatment after the results were issued ……………………………………………………………

....

ACTIONS

74. Sputum sample collected *(Circle)* 1. Yes 2. No

75. If no reason_____________________________

76. Sputum shipped to central Lab *(Circle)* 1. Yes 2. No

77. If no reason_____________________________

78. Sample sent to central lab 1. Spot 2. Early morning

79. Sample on which microscopy and GeneXpert were done

**Microscopy** *(Circle)*	**GeneXpert** *(Circle)*
1. Spot 2. Early morning	1. Spot 2. Early morning

LABORATORY RESULTS (FROM SITE)

80. FM microscopy *(Circle)*	81. GeneXpert *(Circle)*
1. No AFBs seen	1. MTB not detected
2. Positive 1+	2. MTB detected very low
3. Positive 2+	3. MTB detected low
4. Positive 3+	4. MTB detected medium
5. Positive 4 AFBs/100 fields	5. MTB detected high
	6. MTB detected Rif Resistance

Responsible Officer: ………………………………………………………………………………………………

Date:………………………………….. *Sign…………………………………………………*
